# Comparative analyses on epidemiological characteristics of dengue fever in Guangdong and Yunnan, China, 2004–2018

**DOI:** 10.1186/s12889-021-11323-5

**Published:** 2021-07-13

**Authors:** Yujuan Yue, Qiyong Liu, Xiaobo Liu, Haixia Wu, Mingfang Xu

**Affiliations:** 1 State Key Laboratory of Infectious Disease Prevention and Control, National Institute for Communicable Disease Control and Prevention, Chinese Centre for Disease Control and Prevention, Beijing, 102206 People’s Republic of China; 2 Shandong First Medical University & Shandong Academy of Medical Sciences, Tai’an, People’s Republic of China

**Keywords:** Comparative analyses, Epidemiological characteristics, Temporal, Spatial, Demographic, Guangdong, Yunnan

## Abstract

**Background:**

In China, Guangdong and Yunnan are the two most dengue-affected provinces. This study aimed to compare the epidemiological characteristics of dengue fever in Guangdong and Yunnan during 2004–2018.

**Methods:**

Descriptive analyses were used to explore the temporal, spatial, and demographic distribution of dengue fever.

**Results:**

Of the 73,761 dengue cases reported in mainland China during 2004–2018, 93.7% indigenous and 65.9% imported cases occurred in Guangdong and Yunnan, respectively. A total of 55,970 and 5938 indigenous cases occurred in 108 Guangdong and 8 Yunnan counties, respectively during 2004–2018. Whereas 1146 and 3050 imported cases occurred in 84 Guangdong and 72 Yunnan counties, respectively during 2004–2018. Guangdong had a much higher average yearly indigenous incidence rate (3.65 (1/100000) vs 0.86 (1/100000)), but a much lower average yearly imported incidence rate (0.07 (1/100000) vs 0.44(1/100000)) compared with Yunnan in 2004–2018. Furthermore, dengue fever occurred more widely in space and more frequently in time in Guangdong. Guangdong and Yunnan had similar seasonal characteristics for dengue fever, but Guangdong had a longer peak period. Most dengue cases were clustered in the south-western border of Yunnan and the Pearl River Delta region in Guangdong. Most of the imported cases (93.9%) in Guangdong and Yunnan were from 9 Southeast Asian countries. Thailand, Cambodia, and Malaysia imported mainly into Guangdong while Myanmar and Laos imported into Yunnan. There was a strong male predominance among imported cases and an almost equal gender distribution among indigenous cases. Most dengue cases occurred in individuals aged 21–50 years, accounting for 57.3% (Guangdong) vs. 62.8% (Yunnan) of indigenous and 83.2% (Guangdong) vs. 62.6% (Yunnan) of imported cases. The associated major occupations (house worker or unemployed, retiree, and businessman, for indigenous cases; and businessman, for imported cases), were similar. However, farmers accounted for a larger proportion of dengue cases in Yunnan.

**Conclusions:**

Identifying the different epidemiological characteristics of dengue fever in Guangdong and Yunnan can be helpful to formulate targeted, strategic plans, and implement effective public health prevention measures in China.

## Background

Dengue fever, one of the most prevalent mosquito-borne diseases in humans, is mainly transmitted by *Aedes aegypti* and *Aedes albopictus* [1]. There are four distinct serotypes of dengue virus, namely DENV 1–4 [2]. Dengue fever is endemic in more than 100 countries in Southeast Asia, the Americas, Western Pacific, Africa, and Eastern Mediterranean regions, and has evolved from a sporadic disease to a major public health problem as the geographical spread, numbers of cases, and disease severity increased [3]. It is estimated that 390 million people have dengue virus infections with 96 million cases occurring annually, worldwide [1].

Dengue viruses have spread rapidly within countries and across regions in the past few decades. This has resulted in the increased frequency of epidemics and severe dengue disease, hyper-endemicity of multiple dengue virus serotypes in many tropical countries, and autochthonous transmission in Europe and the USA [3]. Prospective cohort studies in Nicaragua and Thailand indicate an annual incidence of dengue virus infection of 6–29% [4, 5]. Dengue activity in Africa has increased substantially, and dengue outbreaks in India and the Eastern Mediterranean region have also progressively increased [6]. Most outbreak-associated dengue cases were reported in the Western Pacific Region, particularly after the year 2010; these cases were primarily identified in China, Singapore, and Malaysia [7].

A total of 655,324 cases and 610 deaths were reported in mainland China from 1978 to 2008, and a total of 52,749 cases and 6 deaths from 2009 to 2014 [8]. A dengue fever outbreak occurred in China in 2014, with 47,127 cases reported, according to Chinese National Notifiable Infectious Disease Reporting Information System (CNNDS) [9]. Dengue fever outbreaks diffused from the southern coastal areas, such as Guangdong (GD) and Hainan to the relatively northern and western areas, including Fujian, Zhejiang, and Yunnan (YN), with shorter outbreak intervals after, than that before the 1990s [10]. The affected regions in China expanded gradually from the coastal provinces to the central provinces over the 10-year period [11].

During 2005–2015, more than 80% of the total dengue cases in mainland China were reported from GD, YN, Fujian, and Zhejiang. Although the overall number of dengue cases were highest in GD, that in YN had increased in recent years to become the highest contributor in 2015 [12]. During 2005–2014, 94.3% of the total indigenous cases in mainland China were from GD versus 3.0% from YN, while 18.3% of the total imported dengue cases were from GD versus 28.8% from YN [11]. Therefore, since these highest reported dengue epidemic cases occurred in both GD and YN, China, it is of great importance to the prevention of dengue fever in mainland China to explore the disease characteristics in these areas. However, comparative analyses of such epidemiological characteristics in these areas are rare. Therefore, this study aimed to compare the epidemiological characteristics of the indigenous and imported dengue cases in GD and YN, using descriptive analyses.

## Methods

### Data collection

Dengue case definition was based on clinical diagnosis as well as laboratory confirmation, in line with the diagnostic criteria and principles of the management of dengue fever (WS 216–2001 and WS 216–2008, before and after 2008, respectively; Table 1) [11, 13, 14].

**Table 1:** Summary of diagnosis criteria and classification for dengue [11]

Variable	Diagnostic Criteria and Principle of Management of Dengue Fever	Diagnostic Criteria for Dengue Fever
Issued by	Chinese Ministry of Health	Chinese Ministry of Health
Date issued	23 November 2001	28 February 2008
Date enforced	1 May 2002	1 September 2008
Epidemiologic Linkage	1.1 Living in or travel to a dengue endemic country/region or presence at location with ongoing outbreak within previous 15 days of dengue-like illness, and reported being bitten by mosquito within 5–9 days of illness onset.	1.1 Travel to a dengue endemic country/region within previous 14 days of dengue-like illness. 1.2 Around the place of residence or place of work (e.g. 100 m radius), there have been dengue case(s) within one month.
Clinical description	2.1 Sudden onset, chills and fever (39–40 °C within 24-36 h, a small number of patients showed a biphasic fever), with symptoms such as fatigue, nausea and/or vomiting. 2.2 Aches and pains (e.g., headache, retro-orbital pain, joint pain, myalgia, arthralgia). 2.3 Flushed skin on face, neck and chest, and conjunctival congestion. 2.4 Superficial lymphadenopathy. 2.5 Measles-like rash, scarlatiniform rash, and/or petechiae in the limbs, trunk, head and face in the course of illness (days 5–7); itching; no scaling; continued 3-5d. 2.6 Encephalitis, encephalopathy, or meningitis-like neurological disorders. 2.7 Bleeding tendency (tourniquet test positive): occurs in the course of illness (days 5–8) with gingival bleeding, nose bleeding, gastrointestinal bleeding, subcutaneous hemorrhage, hematuria, hemoptysis, and vaginal bleeding, and/or chest and abdominal cavity bleeding, etc. 2.8 Multiple organ bleeding. 2.9 Liver enlargement. 2.10 Shock.	2.1 Sudden onset, fever (39–40 °C within 24-36 h, someone shows biphasic fever); severe headache, retro-orbital pain, myalgia, arthralgia and fatigue; flushed skin on face, neck and chest, and conjunctival congestion, etc. 2.2 Rash: measles-like rash, scarlatiniform rash, and/or needle-like hemorrhagic rash in the limbs, trunk, head and face in the course of illness (days 5–7); itching; no scaling; continued 3-5d. 2.3 Bleeding tendency (tourniquet test positive): petechia, ecchymoses, purpura and injection site bleeding, or bleeding from the mucous membranes of mouth and nose, gastrointestinal bleeding, hemoptysis, hematuria and vaginal bleeding in the course of illness (days 5–8). 2.4 Massive hemorrhage of gastrointestinal tract, or chest and abdominal cavity bleeding, or intracranial hemorrhage. 2.5 Liver enlargement, pleural or pericardial effusion. 2.6 Shock syndrome: clammy skin, restlessness, rapid and weak pulse and narrow pulse pressure < 20 mmHg (2.7 kPa) and undetectable in blood pressure, oliguria etc.
Diagnosis and Classification	3.1 Thrombocytopenia (< 100 × 109/L). White blood cell count decrease, lymphocytes and mononuclear cell count increase. 3.2 Hematocrit increased more than 20%. 3.3 IgG anti-DENV positive in a serum specimen. 3.4 IgM anti-DENV positive in a serum specimen. 3.5 IgG anti-DENV ≥4-fold rise in titer in paired acute and convalescent serum specimens. The serologic tests included enzyme-linked immunosorbent assay (ELISA), HI, CF, immunofluorescence method (FA/IFA), Dengue blot (DB), and NT. 3.6 Cell culture isolation of DENV by *Aedes albopictus* C6/36 cell or 1–3 day-old newborn mice; or detection of DENV nucleic acid by RT-PCR; or detection of antigens by monoclonal antibodies immunofluorescence (mbAb-FIA) in serum, cerebrospinal fluid (within 5 days of illness course), other body fluid or tissue.	3.1 A total white blood cell count decrease. 3.2 Thrombocytopenia (< 100 × 109/L). 3.3 Hemoconcentration (an increase in hematocrit ≥20% above average for age or a decrease in hematocrit ≥20% of baseline following fluid replacement therapy); hypoproteinemia. 3.4 IgG or IgM anti-DENV positive in a serum specimen. 3.5 Cell culture isolation of DENV by *Aedes* *albopictus* C6/36 cell or 1–3 day-old newborn mice in acute serum, cerebrospinal fluid, blood, or other tissue specimens. 3.6 IgG anti-DENV ≥4-fold rise in titer in paired acute and convalescent serum samples. The serologic tests included ELISA, mac-ELISA, HI, FA/IFA, NT. 3.7 Detection of DENV nucleic acid by RT-PCR or real-time fluorescence quantitative PCR.
Diagnosis and Classification	4.1 Suspected case: a patient with item 1.1, 2.1 and 2.2, and one of item 2.3 to 2.7, as defined above. 4.2 Probable case: a suspected case with item 3.1 in a confirmed outbreak, or a suspected case with item 3.1 and 3.3 in an unconfirmed outbreak or presented as a sporadic case. 4.3 Confirmed case: DF: a probable case with one of item 3.4, 3.5 and 3.6. DHF: a confirmed DF case with item 2.8, 2.9 and 3.2. DSS: a confirmed DHF case with item 2.10.	4.1 Suspected case: a patient with item 1.1 and 2.1, or a patient with item 2.1, 3.1 and 3.2, as defined above. 4.2 Probable case: DF: a suspected case with 1.2, 3.1 and 3.2; or a suspect case with item 2.1, 3.1, 3.2 and 3.4. DHF: a probable case of DF with item 3.2, 3.3 and one of item 2.3 to 2.5. DSS: a probable case of DHF with item 2.6. 4.3 Confirmed case: a probable case with one of item 3.5 to 3.7.

Dengue fever, a vector-borne notifiable disease in China, is reported to Chinese Centre for Disease Control and Prevention (China CDC) through CNNDS. A dengue case report includes information on sex, age, occupation, national code of current address, date of illness onset, remarks, etc. Several occupation categories are reported including farmer, businessman, and student, among others. Daily dengue case reports in GD and YN, China from 1 January, 2004 to 13 December, 2018 were obtained from CNNDS. The vector data from the Chinese administrative divisions that were used for the geographical mapping were obtained from CNNDS. Monthly temperature and monthly rainfall, from 2004 to 2018, were obtained from the Library for Climate Studies of Chinese Meteorological Administration. Gross domestic product (GDP) value in 2018 was obtained from National Bureau of Statistics of China. The population sizes for 2004–2018 were obtained from the Bureaus of Statistics of YN and GD.

### Data processing

Dengue cases were divided into indigenous, imported, and others according to the diagnostic criteria and principles of the management of dengue fever in China. Patients with indigenous dengue fever were defined as those that had not moved out of the local counties (the current addresses), 14 days before the illness onset. Indigenous cases occurred from June to December annually in this study. Imported ones had been to foreign countries or regions where dengue fever was prevalent, 14 days before illness onset. Other cases included those that we were unsure of how to classify. To perform the spatial analysis, dengue cases were aggregated at the county level according to the national codes of the patients’ current addresses, and these codes were geocoded and matched to the county-level administrative boundaries using ArcGIS version 10.5 [15].

### Data analysis

Analyses of temporal and demographic distribution were conducted using IBM SPSS Statistics for Windows, version 24.0 (IBM Corp., Armonk, N.Y., USA). The chi-square test was used to compare the overall discrepancies of dengue cases by sex, age group, and occupation distributions in GD and YN, 2004–2018. Significant statistical differences between groups were reported at *P* ≤ 0.05. Spatial distribution analyses for the dengue cases were conducted using ArcGIS version 10.5 [15].

## Results

Differences in the general information between GD and YN, in China, are shown in Table 2. The population ratio between GD and YN was 2.3, but the area ratio was only 0.5. Compared with YN, GD had a slightly smaller number of districts and counties. The average yearly incidence rate of indigenous dengue fever in GD, 2004–2018, reached 3.65 (1/100000), while that in YN was 0.86 (1/100000). However, the average yearly incidence rate of imported dengue fever in GD, 2004–2018, reached 0.07 (1/100000), while that in YN was 0.44 (1/100000). There were 73,761 dengue cases in mainland China during 2004–2018, among which 93.7% indigenous dengue cases, and 65.9% imported dengue cases occurred in GD and YN, respectively.

**Table 2:** General information comparison between GD and YN, China

	Population in 2010	Area (km^2^)	Administrative divisions	Average yearly incidence rate of indigenous dengue fever in 2004–2018 (1/100000)	Average yearly incidence rate of imported dengue fever in 2004–2018 (1/100000)
GD	104,409,400	177,548	123 counties in 21 cities	3.65	0.07
YN	46,016,000	383,966	129 counties in 16 cities	0.86	0.44

### Comparative analyses of indigenous dengue fever in GD and YN

#### Temporal comparative analyses of indigenous dengue fever

**Fig. 1:**
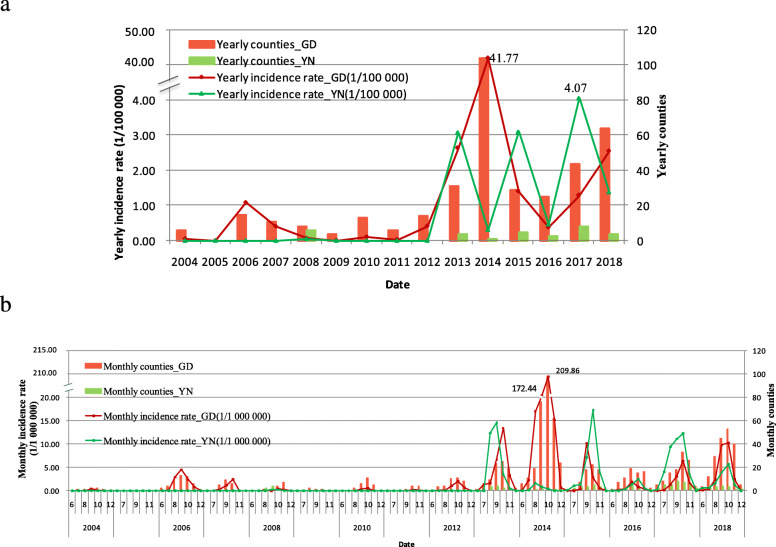
Temporal distribution mapping of indigenous dengue fever in GD and YN, 2004–2018. **a** Yearly indigenous fever. **b** Monthly indigenous fever

There were 55,970 and 5938 indigenous dengue cases in GD and YN during 2004–2018, respectively. Indigenous dengue cases increased in number in recent years (Fig. 1a). The indigenous cases and affected counties both showed a seasonal pattern from July to November (Fig. 1b). No indigenous cases occurred in GD, in 2005. Indigenous cases occurred in YN only in 2008 and 2013–2018. The yearly indigenous incidence rate in YN reached a peak of 4.07 (1/100000) in 2017. In the 2014 dengue fever outbreak in GD, 44,795 indigenous cases, which accounted for 80.0% of the indigenous cases in GD during 2004–2018, were reported, and the yearly indigenous incidence rate was 41.77 (1/100000). There were more indigenous affected counties in GD than that in YN each year in 2004–2018 except in 2005. The monthly indigenous incidence rate and the number of affected counties in GD, in October 2014, reached the highest at 209.86 (1/1000000) and 97, respectively.

#### Spatial comparative analyses of indigenous dengue fever

**Fig. 2:**
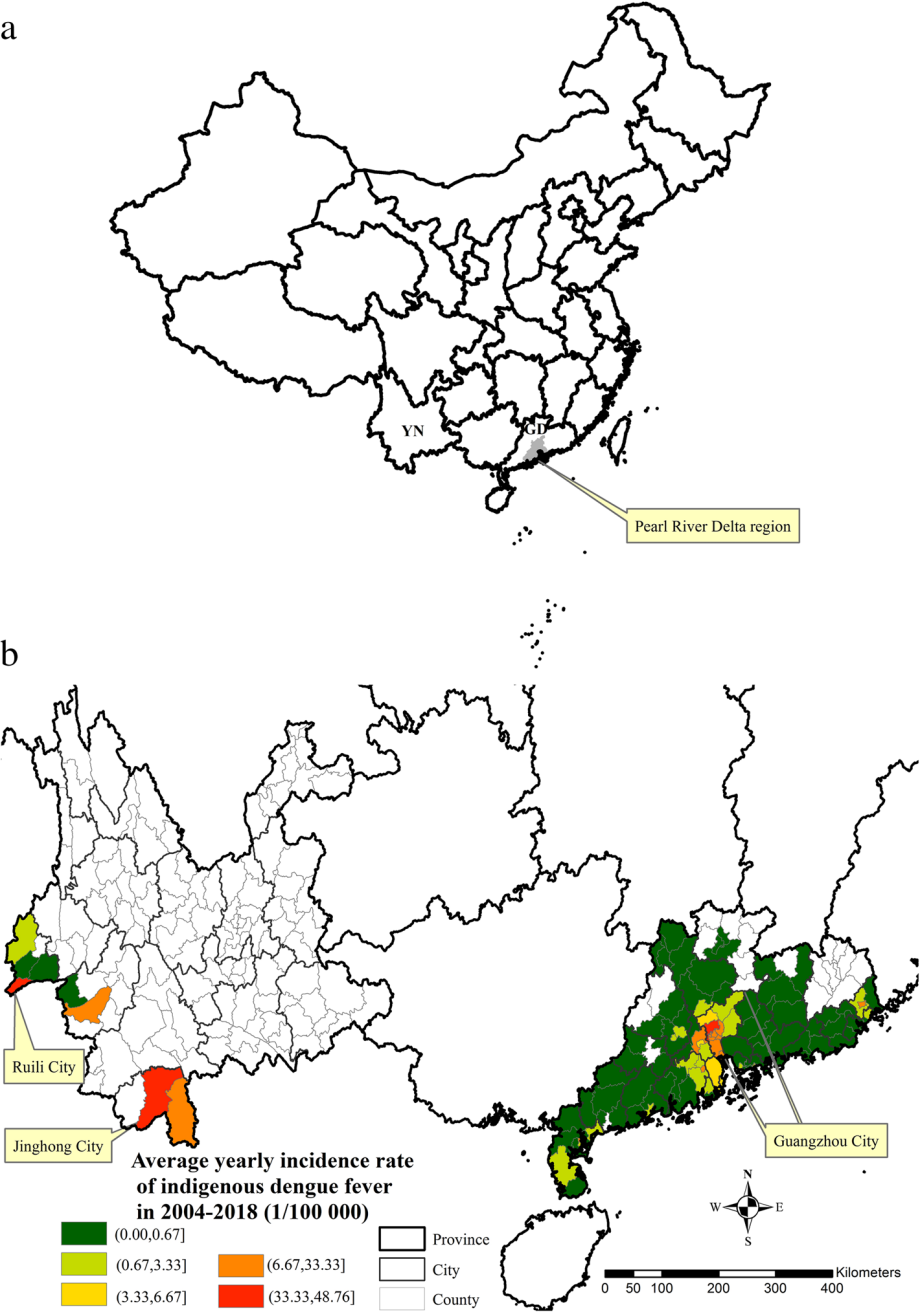
Spatial distribution of indigenous dengue fever in GD and YN, 2004–2018. **a** The study area. **b** Indigenous dengue incidence rate. (Fig. 2 was created using ArcGIS version 10.5 by ourselves. Its license number was SO20160927002)

YN is in south-western China, while GD is in southern China (Fig. 2a). Compared with 108 indigenous affected counties in GD (87.8% of its total number of counties), there were 8 indigenous affected counties in YN (6.2% of its total number of counties). Most indigenous cases in YN were clustered in the south-western border, while those in GD were clustered in the Pearl River Delta region. The top two yearly average indigenous incidence rates in YN were 48.76 (1/100000) in Jinghong City, Xishuangbanna Dai Autonomous Prefecture; and 46.10 (1/100000) in Ruili City, Dehong Dai Jingpo Autonomous Prefecture. Indigenous cases in Jinghong City and Ruili City accounted for 85.1% of indigenous cases in YN, 2004–2018. The top two average yearly indigenous incidence rates in GD were 45.84 (1/100000) in Liwan county, Guangzhou City and 37.06 (1/100000) in Baiyun County, Guangzhou City (Fig. 2b).

#### Demographic comparative analyses of indigenous dengue fever

By gender, the composition of the total indigenous cases in GD differed significantly from that in YN during 2004–2018 (chi-square test = 6.353, *P* = 0.012 [< 0.05], Table 3). There were slightly more female indigenous cases than male indigenous cases both in GD and YN.

**Table 3:** Information comparison of dengue fever by gender in GD and YN, 2004–2018

Gender	Indigenous cases/proportions(%)		Imported cases/proportions(%)	
	GD	YN	GD	YN
Male	27,760/49.6	2843/47.9	765/66.8	1638/53.7
Female	28,210/50.4	3095/52.1	381/33.2	1412/46.3
Total	55,970/100.0	5938/100.0	1146/100.0	3050/100.0

By age group, the composition of the total indigenous dengue cases in GD differed significantly from those in YN during 2004–2018 (chi-square test = 2.202E2, *P =* 0.000 [< 0.05], Table 4). Most indigenous cases occurred in individuals aged 21–50 years, accounting for 57.3 and 62.8% of indigenous cases in GD and YN, respectively. The largest proportions were in individuals aged 21–30 years.

**Table 4:** Information comparison of dengue fever by age group in GD and YN, 2004–2018

Age group	Indigenous cases/proportions(%)		Imported cases/proportions(%)	
	GD	YN	GD	YN
0–10	2661/4.8	287/4.8	8/0.7	294/9.6
11–20	4675/8.4	635/10.7	60/5.2	444/14.6
21–30	12,003/21.4	1350/22.7	379/33.1	842/27.6
31–40	10,267/18.3	1196/20.1	369/32.2	600/19.7
41–50	9782/17.5	1182/19.9	206/18.0	467/15.3
51–60	7539/13.5	710/12.0	83/7.2	258/8.5
61–70	5019/9.0	348/5.9	34/3.0	107/3.5
71-	4024/7.2	230/3.9	7/0.6	38/1.2
Total	55,970/100.0	5938/100.0	1146/100.0	3050/100.0

By occupational categories, the composition of the total indigenous dengue cases in GD differed significantly from that in YN during 2004–2018 (chi-square test = 2.153E3, *P =* 0.000 [< 0.05], Table 5). In GD, the top 4 occupational categories for indigenous cases were among house worker or unemployed (23.3%), retiree, (12.6%) businessman (11.3%), and worker (9.9%). However, in YN, the top 4 occupational categories for indigenous cases were among businessman (24.0%), farmer (13.6%), house worker or unemployed (12.8%), and retiree (9.2%).

**Table 5:** Information comparison of dengue fever by occupation group in GD and YN, 2004–2018

Occupation	Indigenous cases/proportions(%)		Imported cases/proportions(%)	
	GD	YN	GD	YN
Cadre	1740/3.1	278/4.7	102/8.9	49/1.6
Worker	5566/9.9	322/5.4	193/16.8	97/3.2
House worker or unemployed	13,030/23.3	760/12.8	174/15.2	199/6.5
Retiree	7065/12.6	546/9.2	39/3.4	18/0.6
Rural laborer	1046/1.9	149/2.5	16/1.4	75/2.5
Farmer	2957/5.3	807/13.6	31/2.7	1122/36.8
Businessman	6336/11.3	1427/24.0	301/26.3	712/23.3
Student	4287/7.7	480/8.1	58/5.1	384/12.6
Children	1463/2.6	140/2.4	6/0.5	155/5.1
Else	1652/3.0	367/6.2	54/4.7	76/2.5
Unavailable	10,828/19.3	662/11.1	172/15.0	163/5.3
Total	55,970/100.0	5938/100.0	1146/100.0	3050/100.0

### Comparative analyses of imported dengue fever in GD and YN

#### Temporal comparative analyses of imported dengue fever

**Fig. 3:**
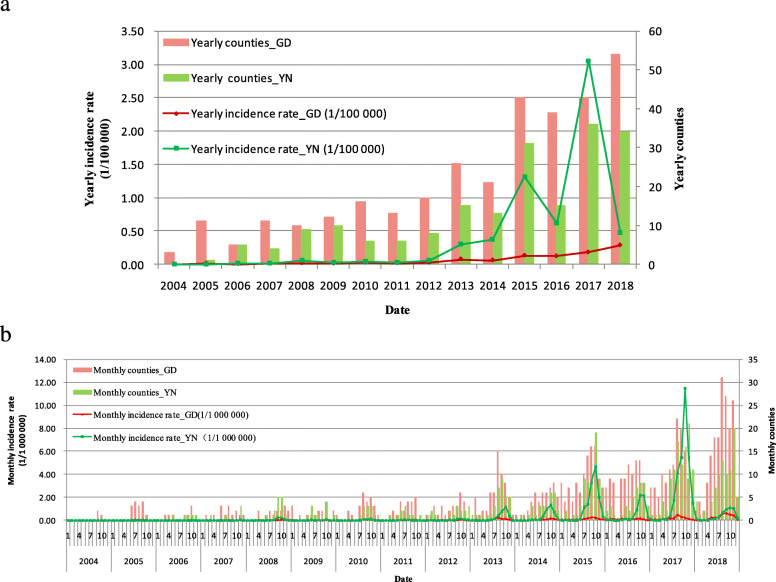
Temporal distribution mapping of imported dengue fever in GD and YN, 2004–2018. **a** Yearly imported fever. **b** Monthly imported fever

There were 1146 and 3050 imported dengue cases in GD and YN during 2004–2018, respectively. The number of imported dengue fever cases increased progressively in recent years (Fig. 3a). No imported case occurred in YN in 2004. There were imported cases in GD throughout the study period (2004–2018). The yearly imported incidence rate reached a peak of 3.06 (1/100000) in YN in 2017. There were 1468 cases in YN in 2017, accounting for 48.1% of its imported cases in 2004–2018. Then the yearly imported incidence rate in GD reached the peak of 0.29 (1/100000) in 2018. There were more imported cases and affected counties in GD than those in YN, yearly, from 2004 to 2018. The monthly imported incidence rate in YN reached the highest at 11.52 (1/1000000) in October 2017. The peak periods of imported cases and affected counties in YN were both from July to December; however, those in GD were from May to December (Fig. 3b).

#### Spatial comparative analyses of imported dengue fever

**Fig. 4:**
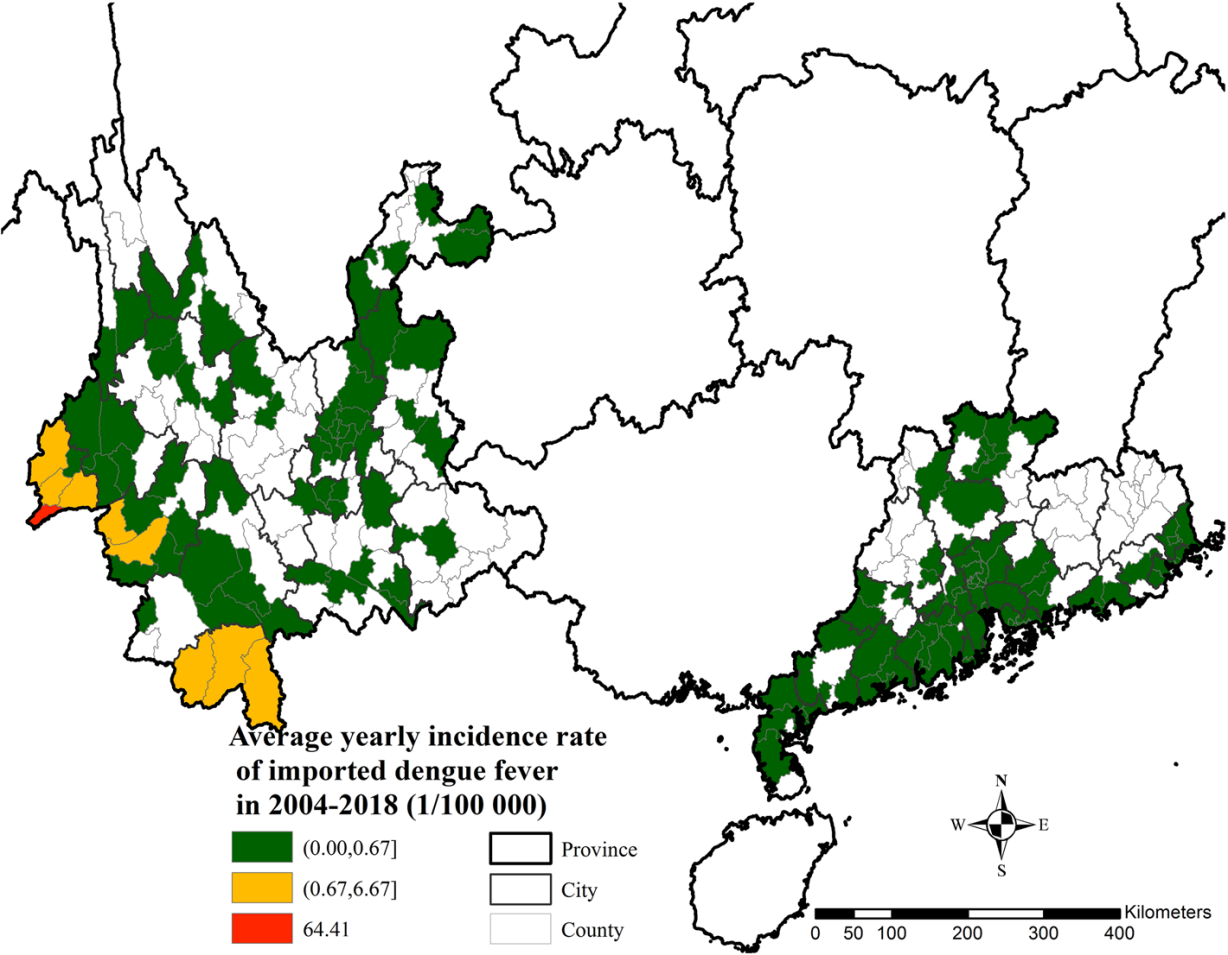
Spatial distribution of imported dengue fever in GD and YN, 2004–2018. (Fig. 4 was created using ArcGIS version 10.5 by ourselves. Its license number was SO20160927002)

Compared with 84 counties in GD (68.3% of its total counties) with imported dengue cases, there were 72 affected counties in YN (55.8% of its total counties). Most imported cases in YN were clustered in the south-western border, while those in GD were clustered in the Pearl River Delta region. The highest average yearly imported incidence rate (64.41 [1/100000]) occurred in Ruili City, Dehong Dai Jingpo Autonomous Prefecture. The average yearly imported incidence rate of more than 0.67 (1/100000) was reported in the border areas of Xishuangbanna City, Lincang City, and Dehong City. The average yearly imported incidence rates in counties in GD were all below 0.67 (Fig. 4).

**Fig. 5:**
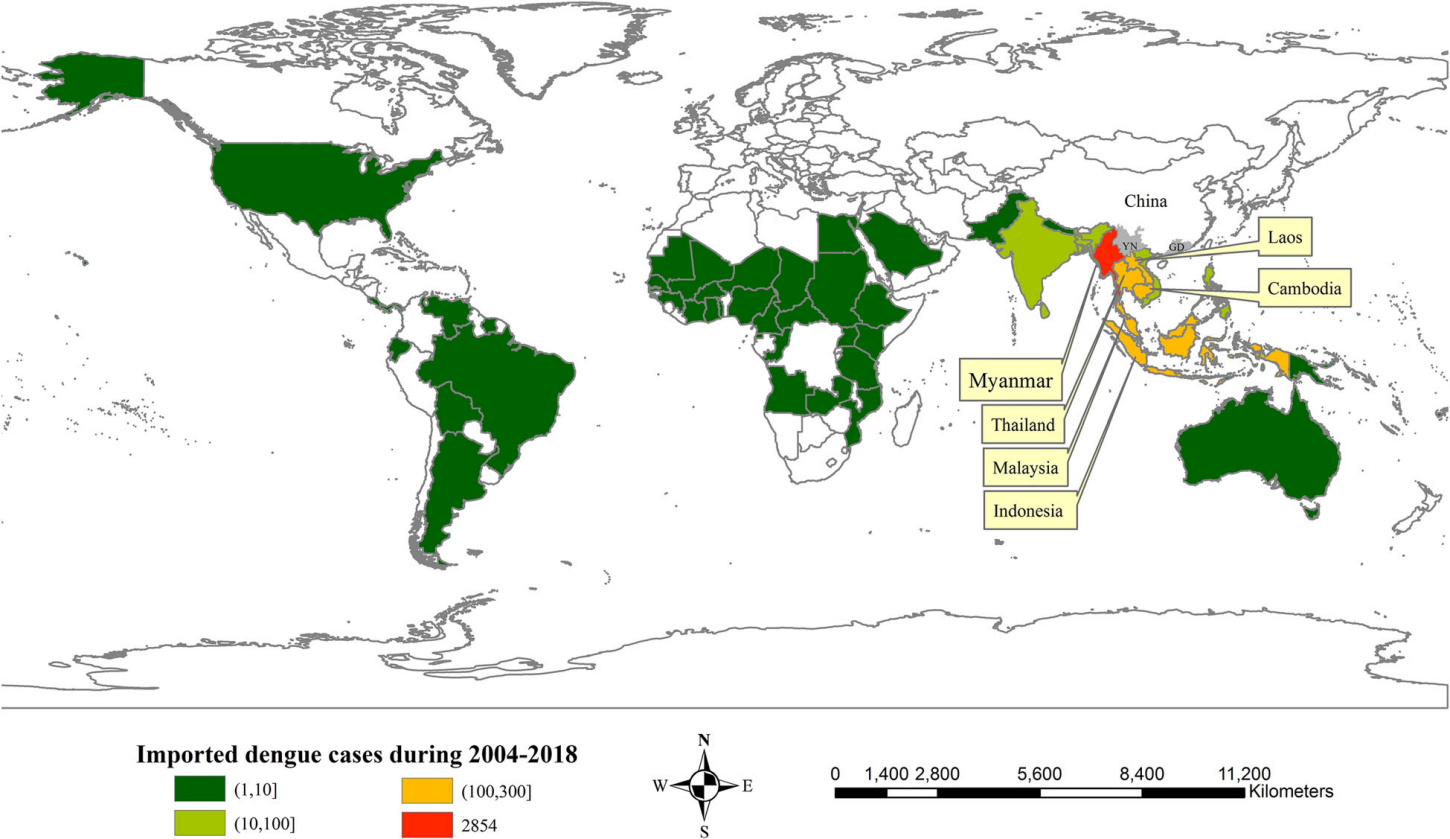
Spatial distribution of the origins of imported dengue fever in GD and YN, 2004–2018. (Fig. 5 was created using ArcGIS version 10.5 by ourselves. Its license number was SO20160927002)

Imported cases in GD could be traced to 42 countries, 19 of which also accounted for the number of imported cases in YN. Nine countries from Southeast Asia (Vietnam, Laos, Cambodia, Thailand, Myanmar, Malaysia, Singapore, Indonesia, and Philippines) were the major contributors, with 3939 imported cases (93.9% of the total imported cases). There were 2854 imported cases from Myanmar (68.0% of the total imported cases). From each of Laos, Thailand, Malaysia, Indonesia, and Cambodia, 100–300 imported cases occurred (Fig. 5). Thailand, Cambodia, and Malaysia were the top 3 sources of imported cases in GD with 220, 171 and 152 cases, respectively. Myanmar, Laos, and Thailand were the top 3 sources of imported cases in YN, with 2793, 122, and 49 cases, respectively.

#### Demographic comparative analyses of imported dengue fever

By gender, the composition of the total imported dengue cases in GD differed significantly from that of YN during 2004–2018 (chi-square test = 57.962, *P* = 0.000 [< 0.05], Table 3). In both provinces, there were more males with imported cases than with the female.

By age group, the composition of the total imported dengue cases in GD differed significantly from that in YN during 2004–2018 (chi-square test = 2.270E2, *P* = 0.000 [< 0.05], Table 4). Most imported cases were in individuals aged 21–50 years, accounting for 83.2% in GD and 62.6% in YN. The largest proportions were in individuals aged 21–30 years.

By occupational categories, the composition of the total imported dengue cases in GD differed significantly from that in YN during 2004–2018 (chi-square test = 1.027E3, *P =* 0.000 [< 0.05], Table 5). In GD, the top 3 occupational categories for imported cases were among businessman (26.3%), worker (16.8%), and house worker or unemployed (15.2%). However, in YN the top 3 occupational categories for imported cases were among farmer (36.8%), businessman (23.3%), and student (12.6%).

## Discussion

During 2004–2018, dengue fever occurred mostly in GD and YN, accounting for 93.7% of indigenous cases and 65.9% of imported cases in mainland China. Dengue fever is closely related to population density and mobility, as well as economic and traffic development [16–19]. GD has a much smaller area but a larger population (Table 2). GD also has a much more developed economy (Table 6). YN is on the border next to Myanmar, where dengue fever was more endemic. Above all, because of the different spatial locations and social conditions, GD had a much higher average yearly incidence rate of indigenous dengue fever, but a much lower average yearly incidence rate of imported dengue fever during 2004–2018. Furthermore, dengue fever occurred more widely in space and more frequent in time in GD.

**Table 6:** GDP comparison in GD and YN, 2018

	GD	YN
GDP (0.1Billion RMB)	97,277.77	17,881.12
The primary industry (0.1Billion RMB)	3831.44	2498.86
The secondary industry (0.1Billion RMB)	40,695.15	6975.44
The third industry (0.1Billion RMB)	52,751.18	8424.82
Per capita GDP (RMB/Person)	86,412	37,136

**Fig. 6:**
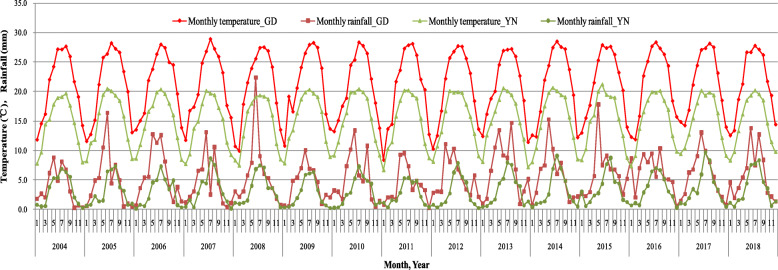
Monthly temperature and monthly rainfall in GD and YN, 2004–2018

GD and YN had similar seasonal patterns for dengue fever, with a longer peak period in GD. They also had similar seasonal patterns for temperature and rainfall (Fig. 6). Dengue fever is closely related to climatic factors such as temperature and rainfall [12, 18, 19]. YN borders a torrid zone, with annual rainfall of 800–1600 mm. The southern region of GD borders a sub-torrid zone, with annual rainfall of more than 1600 mm. These similar climates are responsible for the similar seasonal patterns of indigenous dengue fever [20]. However, imported dengue fever is more related to the economy, population migration, business, and travel, and GD had a much broader imported sources of dengue fever.

High dengue incidence rates in GD occurred in the Pearl River Delta region, especially in Guangzhou City, which is the capital city of GD. High dengue incidence rates in YN occurred along the south-western border adjacent to Myanmar, Laos, and Vietnam, especially in Ruili City and Jinghong City. The 93.9% of the total imported cases in GD and YN occurred from 9 Southeast Asian countries where dengue fever was more endemic [21–24]. Thailand, Cambodia, and Malaysia were the top three sources of imported cases in GD. Myanmar and Laos were the main sources of imported cases in YN. Dengue outbreaks were triggered by imported cases [25]. Thus, both imported and indigenous cases were clustered in similar regions in GD and YN. Therefore, we should focus on the disease prevention and monitoring at the south-western border of YN and the Pearl River Delta region of southern GD; especially Ruili City and Jinghong City in YN, as well as Guangzhou City in GD.

By gender, there was a strong male predominance among imported cases and an almost equal gender distribution for indigenous cases. By age group, most of the dengue cases were among individuals aged 21–50 years (83.2% of the imported cases in GD). This might reflect a population of young working male adults who tend to travel more domestically and regionally and thereby have more exposure risk to dengue [11]. In addition, both indigenous and imported cases occurred across all age groups in GD and YN, which is different from the pattern in other South-eastern Asian countries where most dengue cases occur among children or younger adults [26]. The pattern is most likely because dengue was not endemic in China and the population in China had very low sero-prevalence of dengue antibodies, whereas the populations in endemic countries have higher rates of immunity, especially in adults and the elderly [27–29]. By occupation, there were similarities with the majority affected being house worker or unemployed, retiree, and businessman for indigenous cases, and businessman for the imported cases. Farmers accounted for a larger proportion of dengue cases in YN, which was determined by the industrial structure (Table 5). To address the burden of dengue fever in China, it is necessary to increase the level of knowledge of dengue prevention among people in these occupational categories.

There also existed some limitations to this research. Data quality of dengue case reports from CNNDS should be improved. The remarks in the case report field, described imported sources as foreign countries, used a simple case definition for imported or indigenous cases, or for the process of disease onset and medical treatment. Therefore, the descriptions of the remarks were not standardised. Furthermore, a few remarks were missing. However, dengue cases were divided into indigenous or imported cases mainly based on these remarks. Therefore, there existed some inevitable errors in the division of the cases. Numerous occupational types were unreported; thus, this could have led to missing data on some groups with dengue fever. Above all, these limitations might have influenced the results of this study.

## Conclusion

Dengue fever occurred mostly in GD and YN. This research provided valuable information on the epidemiological characteristics of dengue fever in GD and YN, from 2004 to 2018 by using statistical methods and spatial analysis. GD had a much higher indigenous incidence rate, but a much lower imported incidence rate during 2004–2018. Dengue fever showed similar seasonal patterns in YN and GD. There existed clustering for dengue fever. Most dengue cases in YN were clustered in the south-western border while most cases in GD occurred in the Pearl River Delta region. Thailand, Cambodia, and Malaysia were the top three sources of imported cases in GD. Myanmar and Laos were the main sources of imported cases in YN. There was a strong male predominance among imported cases and an almost equal gender distribution for indigenous cases. Most indigenous and imported cases occurred among 21–50 years old. Similar occupational categories were associated with dengue cases including house worker or unemployed, retiree, and businessman. Furthermore, farmers accounted for a larger proportion of dengue cases in YN. An understanding of the differences in epidemiological characteristics of dengue fever in GD and YN is helpful for the development of targeted, strategic plans, and for implementing effective public health prevention measures.

## Data Availability

Daily dengue case reports were from CNNDS (http://www.chinacdc.cn/). The vector data of Chinese administrative divisions were from CNNDS (http://www.chinacdc.cn/). Monthly average of daily temperature observation data were from Library for Climate Studies of Chinese Meteorological Administration (http://data.cma.cn/). GDP was obtained from National Bureau of Statistics of China (http://www.stats.gov.cn/). Populations in 2004–2018 were from Bureau of Statistics of Yunnan (http://stats.yn.gov.cn/) and Bureau of Statistics of Guangdong (http://stats.gd.gov.cn/).

## Abbreviations

GD: Guangdong
YN: Yunnan
GDP: Gross domestic product
China CDC: Chinese Centre for Disease Control and Prevention
CNNDS: Chinese National Notifiable Infectious Disease Reporting Information System.
